# Phenotypic Characterization of Pectinase‐Producing Alkaliphilic Microbial Isolates From Lake Bogoria, Baringo County, Kenya

**DOI:** 10.1002/mbo3.70058

**Published:** 2025-09-09

**Authors:** Kevin Raymond Oluoch, Edward Kirwa Muge, Maxwell Omondi Onyango, Francis Jakim Mulaa

**Affiliations:** ^1^ Department of Biochemistry, Faculty of Science and Technology, Chiromo Campus, Off Riverside Drive University of Nairobi Nairobi Kenya; ^2^ Faculty of Veterinary Medicine, Chiromo Campus, Off Riverside Drive University of Nairobi Nairobi Kenya

**Keywords:** alkaliphiles, *Bacillus halodurans*, pectinase, phenotypic characterization, soda lakes

## Abstract

Alkaline pectinases are in demand in industrial processes that require the degradation of plant pectins at high pH, for example, removal of pectin stains from fabrics, cutlery, and porcelain; treatment of pectic wastewater; fermentation of coffee, tea, and cocoa; manufacture of poultry and animal feeds, and processing of textiles, and so forth. The present study aimed to (a) screen four alkaliphilic microbial isolates, previously obtained from samples collected around Lake Bogoria (soda lake), Baringo County, Kenya, for alkaline pectinases, and (b) characterize the pectinase‐producers. The screening data revealed that all the isolates were pectinase producers, exhibiting catalytic activities that ranged from 1.4 to 1.6 pectinolytic indices (PI) (primary screening) and 0.04–0.15 U/mL (secondary screening). These isolates' colonies, which featured smooth textures and umbonate elevations, were moist, white, or cream. Their cells were motile, aerobic rods that were Gram‐, catalase‐, and oxidase‐positive. In addition, they a) utilized inositol, sucrose, lactose, and glucose, and b) hydrolyzed starch, pullulan, casein, and gelatin. Furthermore, they grew optimally at pH 10.5, 45°C, and in the absence of NaCl but tolerated growth at higher temperatures (up to 55°C) and saline conditions [up to 12.5% (w/v) NaCl]. No growth was detected at neutral pH. Based on these phenotypic characteristics, the indigenous pectinase‐producing microbial isolates from Lake Bogoria were identified as thermo‐halo‐tolerant obligate alkaliphiles that belonged to the species *Bacillus halodurans*. The alkaline pectinases that they produced can potentially find applications in the fore‐mentioned local industrial processes, if harnessed.

## Graphical abstract

Four alkaliphilic microbial isolates were screened, and all were found to produce alkaline pectinases. All the pectinase‐producers were identified as *Bacillus halodurans.*

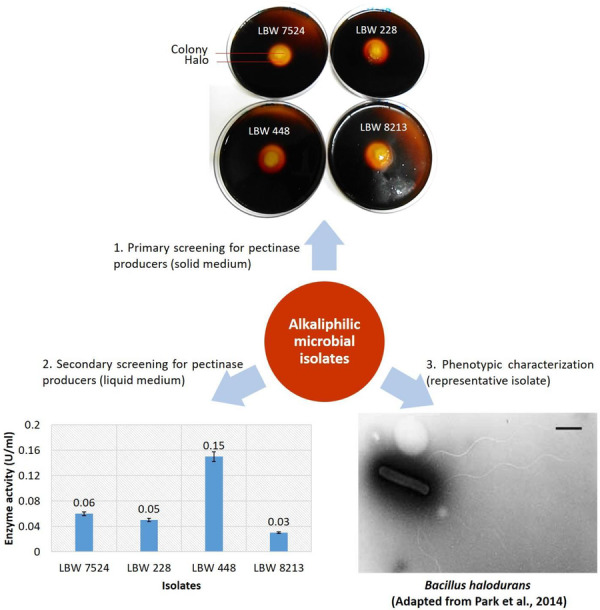

## 1 Introduction

Soda lakes are naturally occurring alkaline environments representing stable and extremely productive aquatic ecosystems. They are generally characterized by (a) high basic pH values around 10 or higher, (b) high concentrations of soda—usually present as natron (Na_2_CO_3_) or trona (Na_2_CO_3_/Na_2_HCO_3_), (c) varying degrees of salinity, and (d) low Mg^2+^ and Ca^2+^ ion concentrations (Boros and Kolpakova 2018). Soda lakes are widely distributed, but due to their inaccessibility, only a few have been explored from the microbiological point of view (Bell 2012). In the East African Rift Valley, there are numerous soda lakes whose pH values range from 8 to 12 and are fed by hot springs, thus providing a potential habitat for thermo‐halo‐alkaliphiles, capable of producing enzymes with novel properties for applications in biotechnology (Jeilu et al. 2022). The microbial populations of these lakes are phylogenetically diverse and include alkaliphiles (organisms that grow at high pH values), e.g., (a) archaea (e.g., halobacteria and methanogens), (b) Gram‐negative bacteria/protobacteria (e.g. *Pseudomonas*, *Halomonas*, sulfur oxidizers, nitrifiers, and anoxygenic phototrophic bacteria), and (c) Gram‐positive bacteria/eubacteria (e.g. *Bacillus* and *Clostridium*) (Grant and Sorokin 2011).

Alkaliphilic microorganisms, especially those that belong to the genus *Bacillus*, have attracted much interest in the past few decades because of their ability to produce extracellular enzymes that are active and stable at high pH values (Mamo and Mattiasson 2016). The unusual properties of these enzymes offer an opportunity for their utilization in industrial processes that demand such extreme conditions, implying that they have the potential to replace the conventional eco‐unfriendly chemicals that are, today used in many industries in developing countries to degrade bio‐polymers in expensive and time‐consuming processes, resulting in the production of subpar goods (Oluoch et al. 2023). Among the industries where these alkaline enzymes are already being utilized include;

- ***Detergent industry:*** proteases, lipases, cellulases, mannanases, amylases and pectinases are added to laundry and automatic/hand dishwasher detergents, to hydrolyze protein‐, fat‐, cellolose‐, mannan‐, starch‐ and pectin‐based food stains, respectively, on garments, china, or cutlery surfaces into smaller bio‐molecules, which can, in turn, be easily reached and removed by the detergent's surface‐active agents (Hede 2020).
- ***Leather industry:*** proteases and lipases are used to de‐hair and de‐grease skin, respectively, during leather production (Lason‐Ryde et al. 2024).
- ***Poultry and animal feed industry:*** proteases, amylases, xylanases, amylases, β‐glucanases, cellulases, xyloglucanase and phytases are added to poultry and animal feeds to i) improve feed digestibility and, as a result, enhance nutritional absorption and ii) release the nutrients generated by the hydrolysis of the polymer and those blocked by it. As a result, less feces is produced, implying more body weight gain and health. (Ozojiofor and Rasheed 2023).
- ***Biofuel industry:*** cellulases are used to hydrolyze cellulosic biomass to fermentable sugars which, are subsequently converted to bioethanol (Naveed et al. 2024).
- ***Beverage industry:*** Pectinases are used to (i) eliminate the pectins found in the cell wall of tea leaves to accelerate the latter's fermentation process, and also destroy the foam‐forming property of instant tea granules thereby enhancing the quality of the tea and consequently, its market value, (ii) hydrolyze the undesired pectin‐rich mucilaginous layer in fresh coffee beans into smaller oligo‐galacturonates that can be easily removed, alongside any other noncellulosic materials (hemicellulose, proteins, natural colorants, etc.) that adhere to it thus, exposing the desired mucilage‐free beans in readiness for further processing, and (iii) degrade cocoa pulp, resulting in good‐quality fermented beans with a chocolate flavor (Haile and Ayele 2022).
- ***Textile industry:*** amylases are used to desize woven cotton while pectinases are used to hydrolyze the pectins found in the cuticular and adjacent primary cell walls of cotton fibers into smaller oligo‐galacturonates that can be easily removed, along with any other noncellulosic materials (waxes, hemicellulose, proteins, natural colorants, etc.) that adhere to it thus, exposing the desired intact soft and smooth hydrophilic cellulosic structures suitable for textile manufacture (Ansell and Mwaikambo 2009; Colombi et al. 2021).
- ***Oil extraction industry:*** pectinases are used to destroy the emulsifying power of pectins, which otherwise interferes with the extraction of oils from a variety of pectin‐rich sources, such as flaxseeds, olives, dates, rape seeds, coconut germs, sunflower seeds, palms, kernels, and citrus peel extracts. The result is the production of premium oils concerning their color, stability, and organoleptic (polyphenolic and vitamin E)‐ and fatty acid‐ contents (Ozojiofor and Rasheed 2023).
- ***Vegetable and food processing industry:*** Proteases, lipases, cellulases, pectinases, and amylases are used to remove proteins, lipids, celluloses, pectins, and starch material from food processing waste waters, rendering them suitable for decomposition by activated‐sludge treatment (Anand et al. 2020; Andler and Goddard 2018).
- ***Pulp and paper industry:*** A combination of pectinases, hemicellulases, cellulases, and ligninolytic enzymes are used to alter the fiber surface bonds around the ink particles on paper, freeing the ink for later removal from the fiber slurry through washing or floatation. This allows for the re‐cycling of waste paper especially from printing press (Anand et al. 2020).
- ***Plant viral research:*** A combination of pectinases and cellulases, are used to hydrolyze plant tissues (especially the phloem) to liberate viruses found therein, for physicochemical and biological research (Anand et al. 2020).
- Whole‐cell alkaliphiles are used to (a) remove toxic sulfur compounds from wastewater and gas streams, and (b) degrade hydrocarbons and other organic and inorganic pollutants (Sorokin et al. 2014).

The Kenyan soda lakes have been extensively studied, where detailed limnological and microbiological investigations have been performed over the years (Grant et al. 1990; Jones et al. 1998). However, there are only a few reports on the study of industrially important prokaryotic populations that inhabit these environments (Bell 2012; Ogonda et al. 2021; Oluoch et al. 2018, 2023, 2024; Vargas et al. 2004). For this reason, there is a need to bio‐prospect for more industrially important microorganism for example, those that produce alkaline enzymes, for use in local industries with an aim to produce high‐quality goods in an eco‐friendly way, as opposed to the (a) the use of eco‐unfriendly and product‐damaging chemicals that are currently used, today, in many developing countries, or (b) costly enzymes that have to be imported. In this article, we report on the phenotypic characterization of indigenous pectin‐degrading microbial isolates from our collection of alkaliphiles.

## 2 Materials and Methods

### 2.1 Screening the Alkaliphilic Microbial Isolates for Extracellular Alkaline Pectinases

Four (4) alkaliphilic microbial isolates (designated as LBW7524, LBW 228, LBW 448, and LBW 8213) were randomly chosen from our collection of 190 alkaliphiles, and subjected to screening for extracellular alkaline pectinases on a) solid (primary screening)‐ and b) liquid (secondary screening)‐ media, respectively, as described in sections 2.2.1 and 2.2.2. ahead. The isolates were previously isolated from soil‐ and water‐ samples obtained from different sites around Lake Bogoria (soda lake) (00° 15’ N and 36° 07’ E), Baringo County, Kenya, using the non‐probability (purposive/selective) sampling method, and stored as 40% (v/v) glycerol stocks at −20°C in the Department of Biochemistry, University of Nairobi, Kenya (Oluoch et al. 2018). Some physicochemical features of this lake include (a) high pH (> 10.3), (b) moderate temperatures (30 ± 4.6°C) and (c) high salinity (70–100 g/L) (McCall 2010).

#### 2.1.1 Primary Screening

The solid screening medium was prepared according to Kelly and Fogarty (1978) but with a slight modification. The medium contained (g/L): pectin from citrus peel, 10; peptone, 3; yeast, 3; CaCl_2_, 3; MnSO_4_.4H_2_O, 0.04; MgCl_2_.6H_2_O, 0.2; K_2_HPO_4_, 1; NaCl, 1.5; Na_2_CO_3_, 10 and agar, 20. The Na_2_CO_3_ was autoclaved separately and then mixed with the remaining medium to bring its pH to 10.5. The medium was poured aseptically into eight (8) petri‐dishes and then allowed to solidify. Four (4) µL of the glycerol stock of each isolate was used to inoculate the medium in duplicate. The plates were incubated at 37°C for 72 h to allow for the growth of the isolates.

One set of the culture plates was flooded with Lugol's iodine solution (1.0 g I, 5.0 g KI, and 330 mL H_2_O), and after 5 min, it was carefully decanted (Hitha and Girija 2014). Pectinase‐producing isolates were detected by the appearance of clear hydrolyzation halos around their respective colonies (Haile et al. 2022). The diameter of the halos and those of their corresponding colonies were measured (mm) and then used to calculate the enzymatic activities (PI) using Equation 1 (Hitha and Girija 2014).

(1) PI value=(Dh)/Dc………….

where: Dh = Diameter of the halo (mm) and Dc = Diameter of colony (mm).

#### 2.1.2 Secondary Screening

The corresponding colonies that were positive for pectinase production following the primary screening process were retrieved from the duplicate culture plates, placed in separate Eppendorf tubes, and then serially diluted using 0.9% (w/v) NaCl solution to obtain cells with an OD of approximately 0.6 (λ 600). Each diluent was used to inoculate 100 mL screening media (prepared as described in section 2.2.1, but without agar) contained in separate 500 mL Erhlenmeyer flasks. The latter were incubated in a thermo‐shaker incubator (Gellenkamp, London, UK) at 37°C and 200 rpm for 48 h to allow the isolates to grow. The cells were harvested by centrifuging at 5000 × *g* and 4°C for 30 min (Hanil Supra 22K‐ Gwangju, South Korea). The cell‐free culture supernatants obtained were considered crude extracts of the enzyme (pectinase).

The activity of the enzyme in the crude extracts was determined according to Oluoch et al. 2018. The enzyme (200 µL) was incubated with 800 µL of 0.5% (w/v) polygalacturonic acid [prepared in 50 mM glycine‐NaOH buffer (pH 10.5)] at 55°C for 10 min. The release of reducing sugars was monitored using the Dinitrosalicylic acid ‐ spectrophotometric assay method (Wang et al. 1997). One unit of pectinase activity was defined as the amount of enzyme that released one µmol of reducing sugars as D‐(+)‐galacturonic acid per minute under the assay conditions. The control was a heat‐inactivated crude enzyme (95°C, 30 min).

### 2.2 Phenotypic Characterization of the Pectinase‐Producing Alkaliphilic Microbial Isolates

The isolates that exhibited extracellular pectinase activities during the two screening processes above were point‐inoculated on nutrient‐agar medium (g/L): Meat extract 1, Yeast extract 2, Peptone 5, NaCl 5, and agar 1.8) [pH adjusted to 10.5 using 20% (w/v) Na_2_CO_3_] and incubated at 37°C for 24 h, unless otherwise stated. The colonies that grew on the plates, if any, were subjected to morphological, biochemical, and physiological characterization, as described ahead:

#### 2.2.1 Morphological Characterization

##### 2.2.1.1 Colony and Cell Characterization

The morphology of the colonies was studied using the naked eye. Among the cultural characteristics assessed were color, texture, elevation, opacity, margin, and consistency (Breakwell et al. 2007).

The morphology of individual cells was studied by (a) mixing a small amount of a 12‐h‐old colony with a drop of saline solution [0.9% (w/v) NaCl] on a microscope slide, (b) covering the mixture with a cover slip, and c) routinely examining the sample under a stereo microscope (Leica EZA D, Cambridge, UK) for cell‐shape, motility, and arrangement. For spore identification, 72‐h‐old colonies were subjected to a similar treatment. The presence of spores is indicated by dark borders within or outside the cells (Cutting et al. 2009). A digital camera integrated into the microscope and a computer running LAS EZ software version 1.8.0 (2000‐2009) was used to capture the microphotographs of these observations.

#### 2.2.2 Biochemical Characterization

##### 2.2.2.1 Oxygen Requirement Test

This was performed according to the method described by Martins et al. (2001). A small amount of each colony was stab‐inoculated in separate 0.6% (w/v) nutrient‐agar medium (pH 10.5) contained in different test tubes. The latter were covered with aluminum foil, incubated at 37°C for 72 h, and then observed for growth. Exclusive growth on the surface of the medium indicated that a particular isolate was an obligate aerobe, while growth in it showed that it was an obligate anaerobe. The presence of growth in and on the surface of the medium indicated that the isolate was a facultative anaerobe (Cason et al. 2019).

##### 2.2.2.2 Gram Staining and Koh‐String Tests

This was carried out according to Gerhardt et al. (1994). A small amount of each colony was suspended in a drop of saline [0.9% (w/v)] solution on different microscope slides and left to dry. The cells were fixed on the slides by passing the latter (cell side up) over a flame. A drop of crystal violet dye was added to stain the heat‐fixed cells (1 min) before rinsing the slides with water. Gram's iodine solution was added to the samples to fix the dye. The samples were then rinsed with water, and 95% ethanol was applied to them (10 s) to remove the unbound crystal violet dye, leaving Gram‐positive bacteria stained purple and Gram‐negative bacteria, if any, colorless. The samples were rinsed with water, counter‐stained with safranin (1 min), and air‐dried. A drop of immersion oil was added to the samples. Finally, the slides were examined, at X‐100 magnification, under a phase contrast microscope (Leica ICC50, Heerbrugg, Switzerland), and the cells were recorded as positive for Gram stain if they appeared purple and Gram‐negative if they were pink.

To complement the Gram‐staining test, the colonies were subjected to the KOH‐string test (Gregersen 1978). A generous amount of each colony was separately mixed with a drop of 3% (w/v) KOH on a microscope slide with the help of a toothpick (applicator). The dense suspension was stirred continuously for 60 s, after which the toothpick was gently lifted and observed. A thick and stringy suspension indicates that the isolate is a Gram‐negative bacterium, while its absence means it is Gram‐positive.

##### 2.2.2.3 Oxidase Test

This was carried out using an oxidase reagent (according to Gordon‐McLeod) according to the manufacturer's instructions (Merck 2021). With the help of a glass rod, each bacterial colony was transferred to a filter paper moistened with the reagent. A change in the color of the colony to purple/black within 10 s due to the oxidation of tetramethyl‐p‐ phenylene diamine (TAPA) to indophenols by intracellular cytochrome C oxidase is a positive oxidase reaction, while the absence of coloration is a negative reaction (Jurtshuk and McQuitty 1976).

##### 2.2.2.4 Catalase Test

A loop full of each colony was stirred with 3% (v/v) H_2_O_2_ on a microscope slide and then observed against a dark background for the evolution of gas (effervescence) (Equation 2), which is indicative of the presence of catalase. On the other hand, its absence indicates the absence of the enzyme (Nandi et al. 2019).

(2) $$2H_{2}O_{2}\;\left(\mathrm{aq}\right)\to {2H}_{2}O\;\left(\mathrm{aq}\right)+O2(g)\ldots \ldots .$$

##### 2.2.2.5 Utilization of Sugars

The colonies were point‐inoculated on modified Horikoshi II solid media (pH 10.5) (Horikoshi 2004). The modification involved substituting the sole carbon source in the medium, that is, starch, with 1% (w/v) inositol, sucrose, lactose, glucose, sorbitol, raffinose, mannitol, and xylose, respectively. The plates were incubated at 37°C for 24 h and then observed for the presence/absence of growth. The presence of growth indicates that the isolate is able to utilize a particular sugar, while its absence implies its failure to do so.

##### 2.2.2.6 Hydrolysis of Carbohydrates and Proteins

###### Starch and Pullulan Hydrolysis

The colonies were point‐inoculated on (a) Horikoshi II solid medium (pH 10.5) (Horikoshi 2004) and (b) a modified version of this medium, containing 1% (w/v) pullulan as the sole carbon source instead of starch. The plates were incubated at 37°C for 72 h and stained with Grams‐iodine solution (Yassin et al. 2021). The hydrolysis of starch or pullulan is detected by the appearance of hydrolysis halos around the colonies, while the absence of the halos indicates a negative test (Ryan et al. 2006).

###### Casein Hydrolysis

This was carried out according to Hussein et al. (2020). The colonies were point‐inoculated on a casein‐nutrient agar medium [pH adjusted to 10.5 using 20% Na_2_CO_3._]. The plate was incubated at 37°C for 72 h and then flooded with 10% (w/v) (NH_4_)_2_SO_4_. The hydrolysis of casein is detected by the appearance of hydrolysis halos around the colonies, while the absence of halos indicates a negative test.

###### Gelatin Hydrolysis

This was performed according to dela Cruz and Torres (2012). A heavy inoculum of each colony was stab‐inoculated in separate nutrient‐gelatin media [(g/L): Peptone 5, beef extract 3, and gelatin 120 (pH adjusted to 10.5 using 20% (w/v) Na_2_CO_3_)] contained in test tubes. The latter were incubated at 37°C for 72 h, after which they were retrieved and placed in a refrigerator (4°C). After 30 min, the tubes were removed, gently tilted, and then observed to determine if the gelatin had been hydrolyzed. Hydrolyzed gelatin will result in a liquid medium even after exposure to cold temperature (4°C), while the uninoculated control medium will remain solid (dela Cruz and Torres 2012).

#### 2.2.3 Physiological Characterization

##### 2.2.3.1 Alkaliphily Test

To determine whether the isolates were obligate alkaliphiles or alkali‐tolerant, they (isolates) were cultured on nutrient‐agar (adjusted to pH 7 with NaOH) at 37°C for 72 h (Martins et al. 2001). Growth at this pH indicated that a particular isolate was alkali‐tolerant, while no growth showed that it was an obligate alkaliphile (Kanekar and Kanekar 2022).

##### 2.2.3.2 Effect of Temperature on the Growth of the Isolates

This was studied by plating out the isolates on a nutrient‐agar medium [pH adjusted to 10.5 using 20% (w/v) Na_2_CO_3_)] and then incubating the plates at 25°C, 37°C, 45°C, 55°C, and 65°C, respectively, for 72 h. Growth at 25°C−45°C indicated a particular isolate was a mesophile (https://www.wordreference.com/definition/mesophilic), while growth at 25°C−60°C showed it was thermo‐tolerant (Flores‐Fernández et al. 2023).

##### 2.2.3.3 Effect of Salinity on the Growth of the Isolates

The isolates were inoculated on nutrient‐agar medium [pH adjusted to 10.5 using 20% (w/v) Na_2_CO_3_)] containing 0, 5, 10, 12.5, and 15% (w/v) NaCl, respectively. This was followed by incubating the plates at 37°C for 24 h. Growth in the presence of > 0%−5% (w/v) NaCl indicated that a particular isolate was a slight halophile, while growth in the presence of > 5%–15% (w/v) salt indicated that it was a moderate halophile. Growth in the presence of 0%−15% (w/v) NaCl indicated that it was halotolerant (Dutta and Bandopadhyay 2022).

#### 2.2.4 Data Analysis

The data for primary screening was obtained after conducting the experiment once on iodine‐stained solid pectin media (pH 10.5). This was done by determining the Dh:Dc ratio of a particular isolate to obtain the enzyme activity (PI). In contrast, the data for secondary screening was obtained after carrying out the experiment, in triplicate, in liquid pectin media, with calculated means, from which the enzyme activity (U/mL) was calculated. The data for characterizing the isolates was obtained after conducting the experiments once, on solid medium, and then studying their phenotypic characteristics visually.

## 3 Results and Discussion

### 3.1 Pectinolytic Activities of the Alkaliphilic Microbial Isolates

The first step in the study was to screen the four (4) alkaliphilic microbial isolates for extracellular alkaline pectinase activity, first on solid medium (primary screening)‐ and then in liquid medium (secondary screening):

#### 3.1.1 Primary Screening

**Figure 1:**
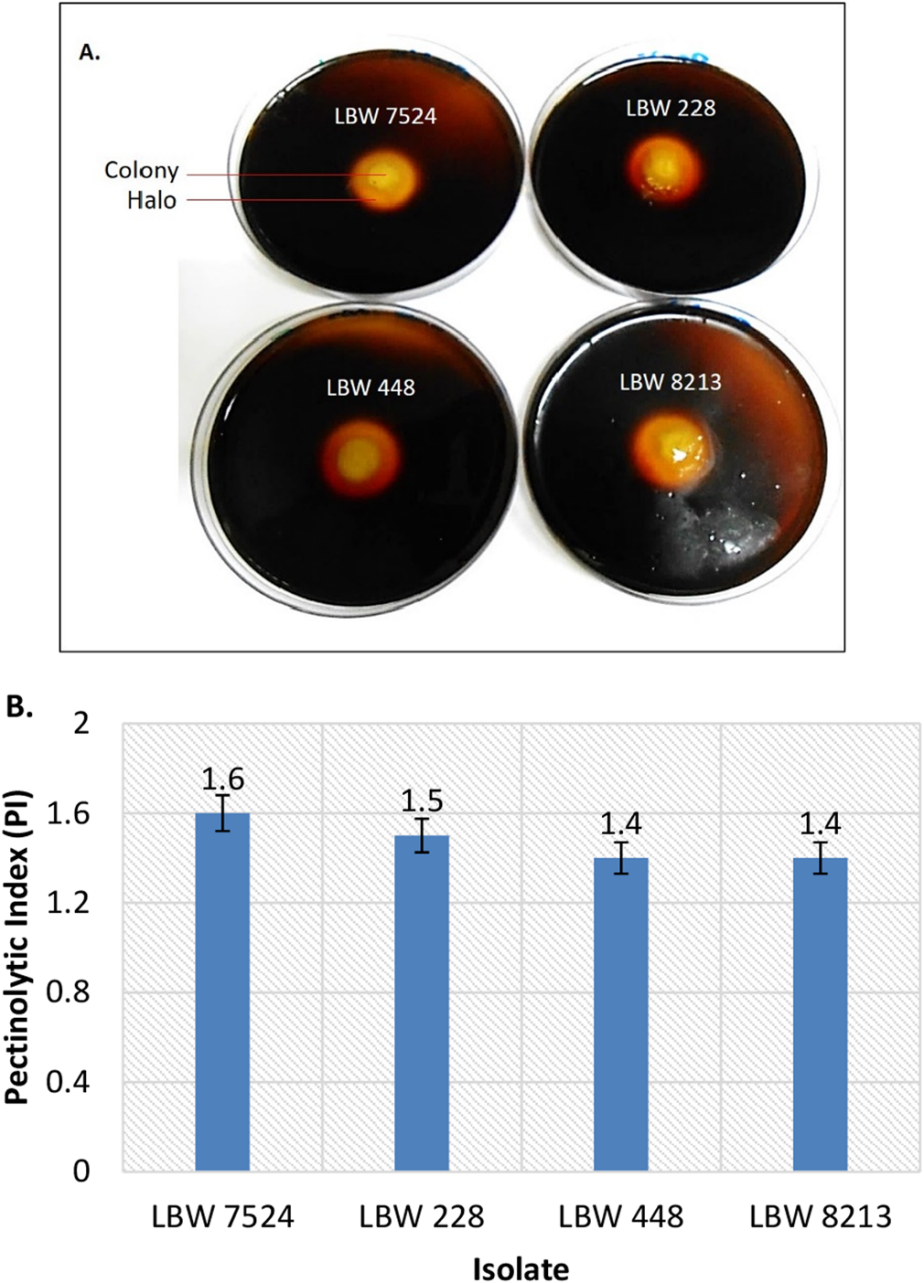
(A) pectin‐stained solid media (pH 10.5) showing hydrolytic halos around the isolates' colonies following 72 h of cultivation at 37°C, and, (B) graph showing the isolate's enzymatic activities (PI) obtained by determining the ratio of the diameter of their halos to that of their corresponding colonies.

All the isolates formed clear hydrolyzation halos around their respective colonies after staining the solid pectin media (pH 10.5) with Lugol's dye solution, indicating the presence of extracellular alkaline pectinase activities (Figure 1A). The enzyme hydrolyzed the pectin around the colonies to prevent the dye from binding to the substrate, thus leading to the decolorization of the dye around the positive colonies (halos) (Hitha and Girija 2014). The size of each hydrolytic halo, obtained by determining the ratio of its diameter to that of its corresponding colony, is directly proportional to the activity of the enzyme (PI) responsible for its formation (Appendix, Table A1).

**Table 1:** Additional biochemical characteristics of the isolates showing how different sugars supported their growth rates on modified Horikoshi II solid medium (pH 10.5) (Horikoshi 2004) over a period of 24 h at 37°C.

Sugar	Isolate			
	LBW 228	LBW 7524	LBW 8213	LBW 448
Inositol	±	±	±	±
Sucrose	++	++	++	+
Lactose	+++	+++	+++	±
Glucose	+++	+++	+++	+++
Sorbitol	++	±	±	+
Raffinose	+++	+++	++++	+++
Mannitol	+++	+++	++++	+++
Xylose	+	+	+	+

*Note:* Key: ± poor growth; + Minimal growth; ++ Moderate growth; +++ High growth; ++++ Very high growth; ‐ No growth.

As seen in Figure 1B, all isolates exhibited more or less similar enzymatic activities, with PI values ranging from 1.4 to 1.6. These results are similar to those reported by other researchers, who went on to show that plate assay method using dye solutions is an easy and rapid method for screening microorganisms for different enzymes (Haile et al. 2022; Kotb et al. 2023; Sinza et al. 2021).

#### 3.1.2 Secondary Screening

**Figure 2:**
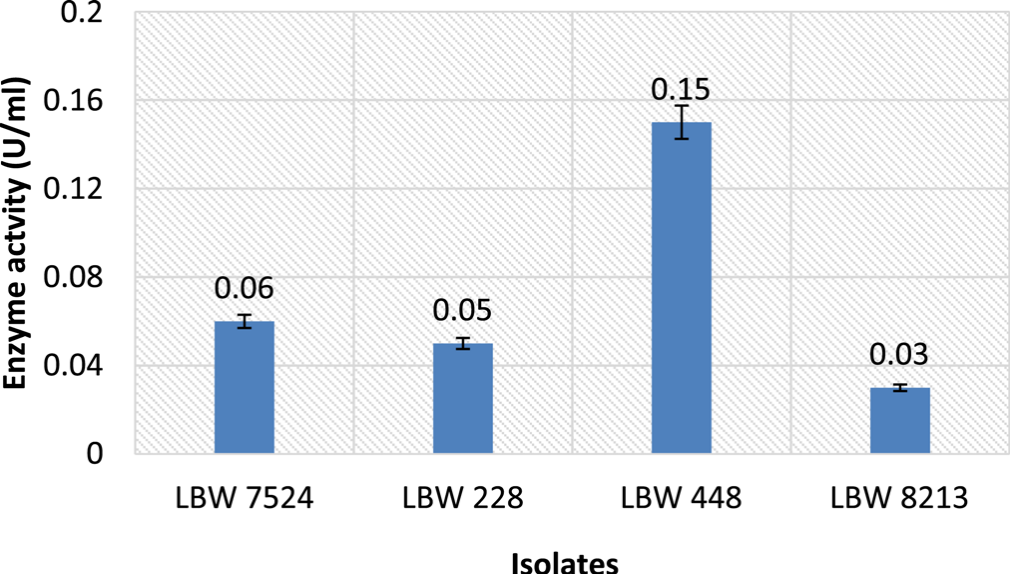
Enzymatic activities of the isolates after 48 h of cultivation at 37°C and pH 10.5 in liquid medium containing pectin as the sole carbon source.

Like on the solid medium (primary screening), all isolates exhibited extracellular alkaline pectinase activities in a liquid medium (secondary screening). The activities ranged from 0.03 to 0.15 U/mL (Figure 2) and were observed to correlate well with those seen on solid medium (Figure 1B). However, the only exception was isolate LBW 448, which displayed an activity that was 3−4 times higher (0.15 U/mL) than those exhibited by the rest of the isolates (Figure 2) but, interestingly, showed a similar activity (1.4 PI) with that of the rest of the isolates on solid medium (Figure 1B). This disparity can be attributed to differences in the (a) uptake of nutrients by the isolate in the two media types and (b) activity detection principle, for example, in the solid medium, the disappearance of the substrate is measured, while in the liquid medium, the product's appearance is measured, as was also reported by Castro et al. (1993).

It is important to note that although the enzymatic activities exhibited by all four (4) isolates in liquid pectin medium are similar to those reported for other alkaline pectinases in research laboratories across the world (Oluoch et al. 2023), they are much lower than those reported for commercial alkaline pectinases for example, 10,000 U/mL for *COENZYME SPA‐5* (http://www.sunsonenzyme.com/Products/Textile/Bio_scouring/2017/0531/198.html). The low activities can be attributed to the un‐optimized culture conditions used to grow the microorganisms (Haile and Ayele 2022). This suggests that the isolates' enzymatic production output can be enhanced, for example, by optimizing their culturing parameters (e.g., medium type and composition, pH, temperature, incubation time, etc.) to help meet the industrial threshold. Alternatively, the activity may be improved by expressing the enzyme in a heterogeneous host, as is the case with some commercial enzymes (Flores‐Fernández et al. 2023). If harnessed in terms of production, these locally produced alkaline pectinases can find applications in diverse local Kenyan industries or institutions and thus, replace the environmentally harmful chemicals that are, today, used therein, to degrade plant pectins in expensive and time‐consuming processes, resulting in the production of subpar goods (Oluoch et al. 2023). Among the industries where they can find applications include; (a) African Fresh Produce EPZ Ltd, Machakos (food industry), (b) Unga Holdings (K) Ltd, Nairobi, and Ruiru Feeds (K) Ltd, Ruiru (feed industry) (c) Thika Textile Mills, Thika, and Rivatex (EA) Ltd, Eldoret (textile industry), (d) Pan African Paper Mills (K) Ltd, Webuye (pulp and paper industry), (e) Unilever Tea (K) Ltd., Kericho, and African Roasters EPZ Ltd., Machakos (Beverage industry), and (f) Department of Plant Science and Crop Protection, University of Nairobi (plant research), and so forth. If this materializes, it can replace the environmentally harmful chemicals that are, today, largely used to degrade plant pectins in expensive and time‐consuming industrial processes, resulting in the production of subpar goods, or even help save our foreign reserves (Oluoch et al. 2023).

### 3.2 Phenotypic Characteristics of the Pectinase‐Producing Alkaliphilic Microbial Isolates

Microbial morphological, biochemical, and physiological characteristics are the most important factors to consider when determining their identity in a given ecological niche (Al‐awadhi et al. 1989). Therefore, the next step in this study was to identify the four Lake Bogoria pectinase‐producing alkaliphilic microbial isolates based on these phenotypic parameters.

#### 3.2.1 Morphological Characteristics

**Figure 3:**
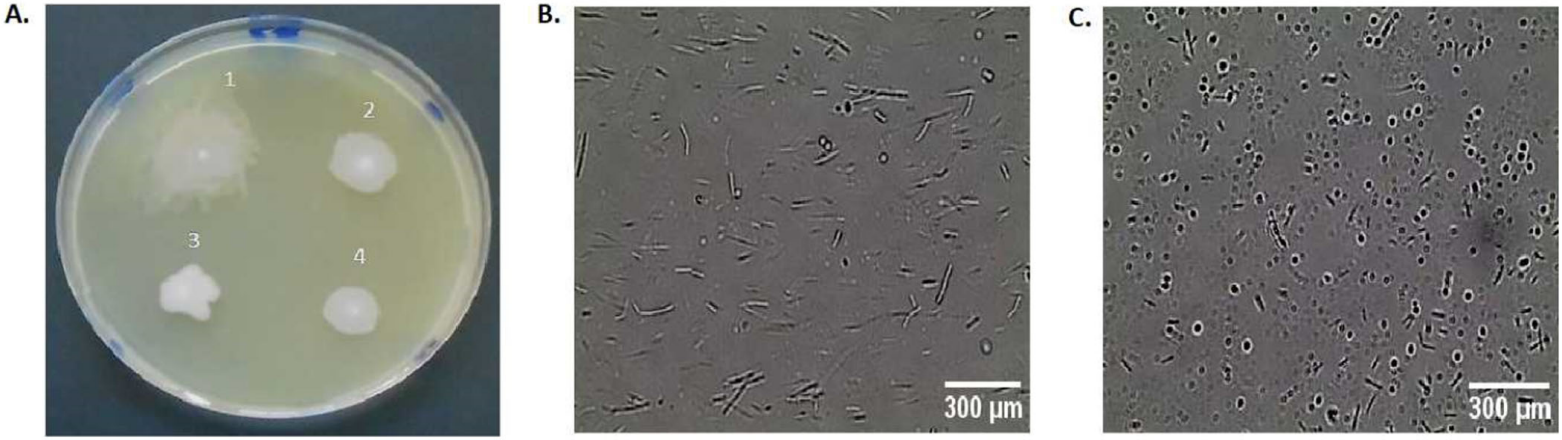
Morphological characteristics of the alkaliphilic microbial isolates grown on nutrient‐agar medium (pH 10.5) at 37°C for various periods of time: (A) colony morphology of 24‐h‐old isolates (1 = isolate LBW 7524; 2 = isolate LBW 228; 3 = isolate LBW 448 and 4 = isolate LBW 8213), (B) cell morphology of a 12‐h‐old representative isolate (LBW 448), and (C) 72‐hour‐old representative isolate (LBW 448) (Magnification ×100).

The colonies of all the isolates were 9 mm when grown on nutrient agar medium at 37°C for 24 h, except for isolate LBW 7524 which was 5 mm (Appendix Table AI). In addition, they were white with smooth, moist textures that were elevated in an umbonate fashion, except for isolate LBW 7524, which was cream (Figure 3A). Furthermore, they had an opaque and glistening appearance and serrated margins, except for isolate LBW 228, which had an undulated margin (Figure 3A). On the other hand, their cell morphology showed that they were motile rods that existed as either single or short chains after 12 h of cultivation but sporulated upon extending the growth period to 72 h (Figure 3B,C). Spore formation was ascribed to nutrient depletion in the medium after an extended cultivation period (Koopman et al. 2022). Among the microorganisms shown to exhibit these growth characteristics are bacteria (Remize 2017), thus, confirming that all the alkaliphilic microbial isolates in the present investigation belong to this group of prokaryotic cells.

#### 3.2.2 Biochemical Characteristics

**Figure 4:**
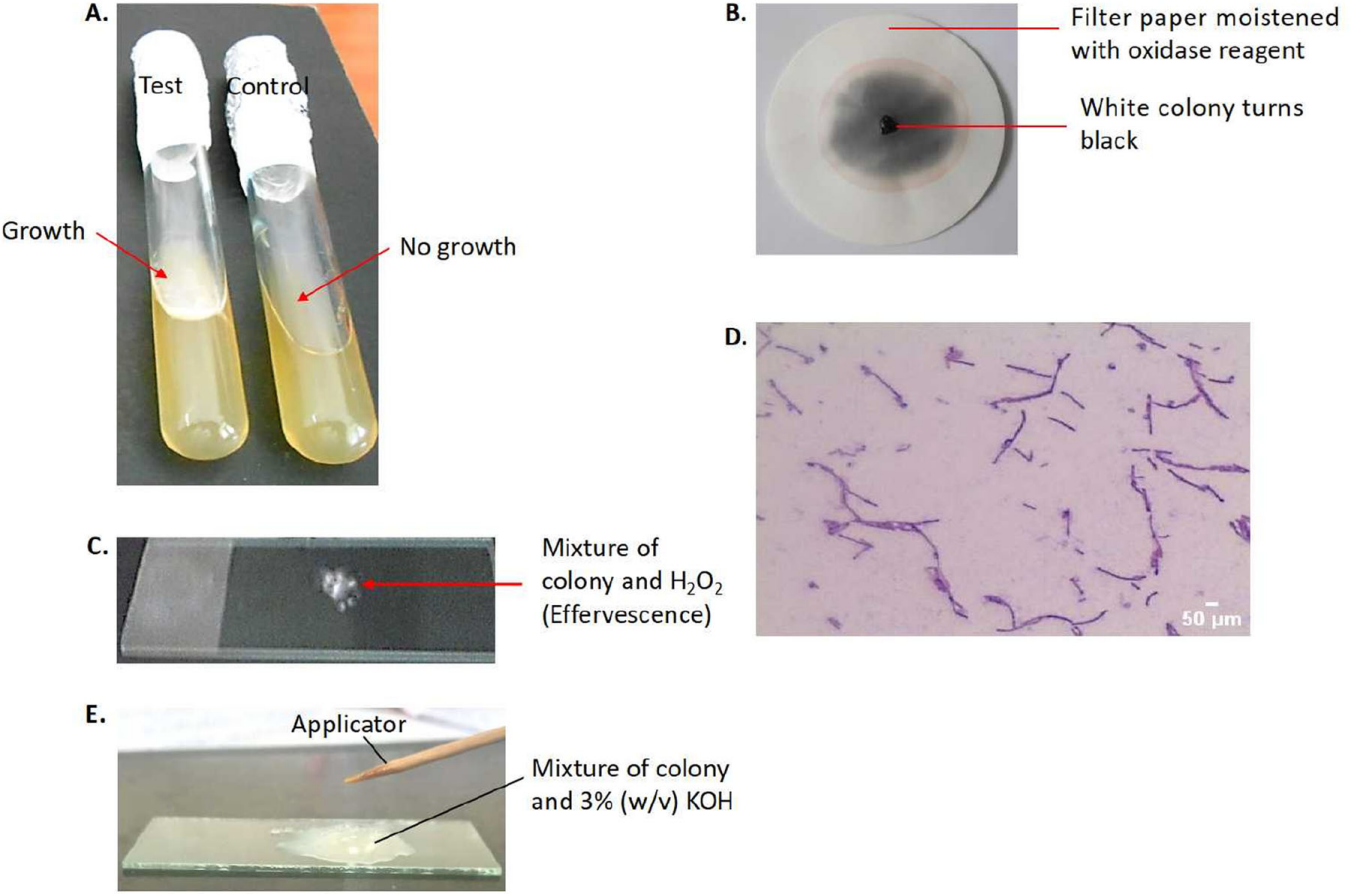
Some biochemical characteristics of a representative isolate, LBW 448, used in the study: (A) Oxygen requirement test, (B) Oxidase test, (C) Catalase test, (D) Gram‐staining, and (E) KOH‐string test. The isolates that were used in these various tests were 24 h old cultures grown on solid nutrient‐agar medium at 37°C, except for those used in the oxygen requirement test, which were 72 h old.

The oxygen requirement test showed that all four (4) isolates were motile and also required oxygen for growth (aerobes). This is because they migrated from the inside of the solid nutrient medium to its surfaces, via the inoculation channels, where they thrived. (Figure 4A). In addition, the oxidase test revealed that they were all positive for cytochrome c oxidase (aerobes) because their colonies turned black with the addition of the oxidase reagent, (Figure 4B) (Shields and Cathcart 2013). This result is consistent with that obtained for the oxygen requirement test above, confirming that all the isolates were aerobic (Figure 4A). Furthermore, the isolates were positive for catalase as evidenced by the formation of effervescence when their colonies were mixed with 3% (v/v) H_2_O_2_ (Figure 4C). Moreover, the Gram‐staining test revealed that they were all Gram‐positive bacteria since, their peptidoglycan cell walls maintained the crystal violet‐iodine complex, giving them a violet color [Figure 4D] (Sharma et al. 2023). Since the standard Gram‐staining method has been demonstrated to be unreliable, the KOH‐string test was used to classify the isolates according to differences in their cell wall groups (Arthi et al. 2003). None of the isolates formed a mucoidal string‐like structure in 3% (v/v) KOH, confirming they were indeed Gram‐positive bacteria (Figure 4E). Bacteria that have been shown to display these biochemical characteristics include those that belong to the Genus Bacillus (Ash et al. 1991; dela Cruz and Torres 2012; Nielsen et al. 1995). This confirms that all the four (4) alkaliphilic bacterial isolates in the present study belong to this Genus.

**Figure A1:**
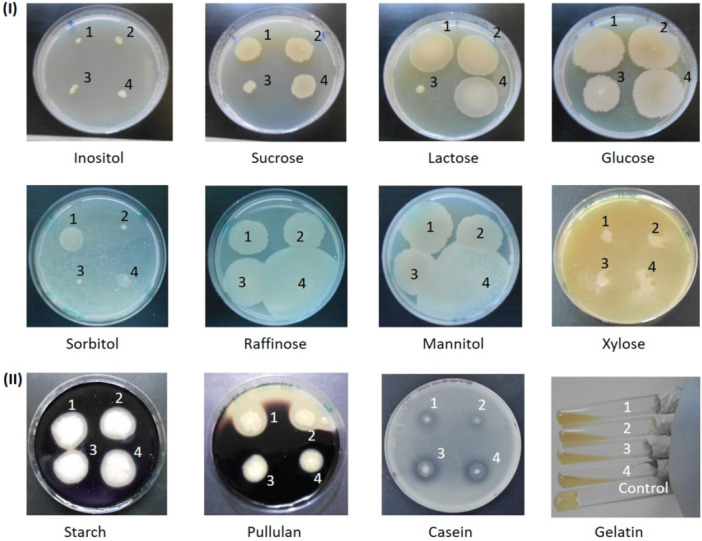
Biochemical characteristics of the isolates showing: (I) how they utilized a variety of sugars to support their different growth rates on modified Horikoshi II medium over a period of 24 h at 37°C (isolate 1 = LBW 228, 2 = LBW 7524, 3 = LBW 8213 and 4 = LBW 448), and (II) hydrolysis halos around their respective 72 h old colonies grown on starch, pullulan, and casein solid media at 37°C, followed by (a) staining of the starch and pullulan media with Gram's iodine solution, and precipitating the casein medium with (NH_4_)_2_S0_4_. and tilting the tubes containing gelatin. Also shown is the hydrolysis of gelatin by the 72 h old isolates, depicted by its liquefaction after tilting the test tubes.

Subsequent biochemical analyses showed that most of the isolates could utilize diverse sugars to support their varying growth rates [Table 1 and Figure A1 (I)]. In addition, the isolates hydrolyzed starch, pullulan, casein, and gelatin (Table 2), as evidenced by the (a) formation of hydrolysis halos around their respective colonies on solid media, and (b) liquefaction of gelatin [Figure A1 (II)] (dela Cruz and Torres 2012). This shows that all four isolates were producers of amylases, pullulanases, caseinases, and gelatinases. It has been demonstrated that several alkaliphilic Bacillus sp. nov, such as Bacillus alcalophilus, Bacillus agaradhaerens, Bacillus halmapalus, Bacillus halodurans, and Bacillus pseudoalcalophilus, utilize these sugars, and also hydrolyze these carbohydrates and proteins (Nielsen et al. 1995). This suggests that the alkaliphilic microbial isolates in this study may be among these bacterial species.

**Table 2:** More biochemical characteristics of the isolates showing their ability to hydrolyze different carbohydrates and proteins.

Sugar/Protein	Isolate			
	LBW 228	LBW 7524	LBW 8213	LBW 448
Starch	+	+	+	+
Pullulan	+	+	+	+
Casein	+	+	+	+
Gelatin	+	+	+	+

*Note:* Key: + Hydrolysis of the carbohydrate and protein.

#### 3.2.3 Physiological Characteristics

**Figure A2:**
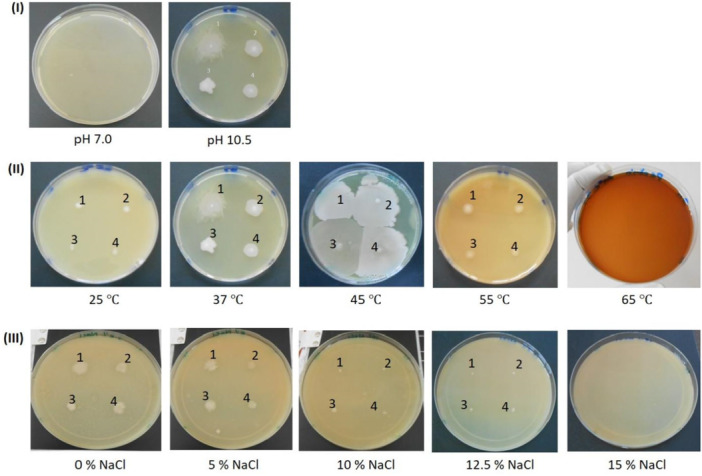
Physiological characteristics of the isolates: Effect of pH (alkaliphily test) (I), temperature (II) and salinity (III) on the growth of the isolates. Isolate 1 = LBW 228, 2 = LBW 7524: 3 = LBW 8213, and 4 = LBW 448. All the isolates were grown on nutrient‐agar medium (pH 10.5) at 37°C for 72 h, except for the (a) alkaliphily test, where an additional nutrient‐agar medium (pH 7) was included, b) growth temperature, where additional temperatures (25°C, 45°C, 55°C, and 65°C) were included, and c) growth salinity, where additional salt concentrations (5%, 10%, 12.5%, and 15% (w/v) NaCl were included.

Among the phenotypic characteristics of microbial isolates, growth pH‐, temperature‐, and tolerance to salt (NaCl)‐ are considered crucial in determining the identity and, hence, diversity of alkaliphilic *Bacillus* strains in a given ecological niche (Nielsen et al. 1995). Thus, the isolates in the study were subjected to these physiological characterizations to identify the species they belong to. The alkaliphily test revealed they could grow at pH 10.5 but not at neutral pH [Table 3, Appendix A2 (I)], confirming that they belonged to a group of alkaliphiles referred to as obligate alkaliphiles (Gundala et al. 2013). Furthermore, they demonstrated thermo‐ and halo‐tolerance by growing up to (a) 55°C (optimal 45°C) [Table 3, Figure A2 (II)] (Flores‐Fernández et al. 2023) and (b) 12.5% (w/v) NaCl [optimum 0%−5% (w/v) NaCl] [Table 3, Figure A2 (III)] (Albdaiwi et al. 2024). These physiological growth characteristics are identical to those exhibited by *Bacillus halodurans* (Nielsen et al. 1995) and, therefore, confirm that all four (4) alkaliphilic microbial isolates in the present study belong to this species. *Bacillus halodurans* is a motile, rod‐shaped, aerobic thermo‐halo‐tolerant obligate alkaliphile that forms endospores and is Gram‐, catalase‐, and oxidase‐positive (Nielsen et al. 1995). It is a common bacterial species that inhabits soda lakes within the Kenyan Rift Valley area and the hot springs surrounding them (Duckworth et al. 1996). The limited number of bacterial species obtained in this study can be attributed to the sampling method and limited isolation conditions that we previously employed in the field and laboratory, respectively (Oluoch et al. 2018).

**Table 3:** Physiological characteristics of the alkaliphilic microbial isolates grown on nutrient‐agar medium (pH 10.5) at 37°C for 72 h. Exceptions were: (a) alkaliphily test, where an additional nutrient‐agar medium (pH 7) was included, (b) growth temperature, where additional temperatures (25°C, 45°C, 55°C, and 65°C) were included, and c) growth on saline conditions, where additional salt concentrations (5%, 10%, 12.5%, and 15% (w/v) NaCl were included.

Parameter evaluated		Isolate			
		LBW 228	LBW 7524	LBW 8213	LBW448
pH	7.0	—	—	—	—
	10.5	+++	+++	+++	+++
Temperature (°C)	25	+	+	+	+
	37	+++	+++	+++	+++
	45	++++	++++	++++	++++
	55	++	++	++	++
	65	—	—	—	—
NaCl (%)	0	+++	+++	+++	+++
	5.0	++	++	++	++
	10.0	+	+	+	+
	12.5	±	±	±	±
	15	—	—	—	—

*Note:* Key: ± poor growth; + Minimal growth; ++Moderate growth; +++ High growth; ++++ Very high growth; ‐ No growth.

## 4 Conclusions and Recommendation

The study demonstrates that all four pectinase‐producing microbial isolates from Lake Bogoria, Baringo County in Kenya, were thermo‐ halo‐ tolerant obligate alkaliphiles that belonged to the species *Bacillus halodurans*. Although the enzymatic titers that these bacteria produced were quite low (0.04−0.15 U/mL) compared to those reported for commercial alkaline pectinases, [e.g., COENZYME SPA‐5 (10,000 U/mL)], they can find applications in many industries, if harnessed in terms of production. Among the local industries or research institutes where they can find applications include the (a) food, (b) feed, (c) textile, (d) pulp and paper, (e) beverage, and (f) plant virus research institutions, and so forth. However, their potential in these applications must be demonstrated under optimized operating conditions before scaling their production levels to pilot‐ and industrial‐ scales in that order (Sunsonzymes 2024).

## Author Contributions

**Maxwell Onyango Omondi:** methodology (supporting), investigation (lead), formal analysis (lead), data curation (support), validation (lead). **Kevin Raymond Oluoch:** conceptualization (lead), methodology (lead), investigation (lead), data curation (lead), supervision (lead), resources (lead), writing – original draft (lead), writing – review and editing (equal). **Edward Kirwa Muge:** conceptualization (lead), methodology (support), supervision (support), writing – review and editing (equal). **Francis Jakim Mulaa:** conceptualization (lead), methodology (support), supervision (support), writing – review and editing (equal).

## Ethics Statement

The authors have nothing to report.

## Conflicts of Interest

The authors declare no conflicts of interest.

## Data Availability

This published article includes all the data generated or analyzed during this study.

## 1

**Table A1:** The pectinase activities of the four isolates (PI), calculated from the ratios of the diameters of their hydrolytic halos to that of their corresponding 72 h old colonies grown on modified Kelly & Fogarty medium at 37°C.

Isolate	Diameter of colony (mm)	Diameter of hydrolytic halo (mm)	Size of hydrolytic halo (PI)
LBW 7524	5	8	1.6
LBW 228	9	13.5	1.5
LBW 448	9	13	1.4
LBW 8213	9	13	1.4
